# Levosimendan to Facilitate Weaning From Cardiorespiratory Support in Critically Ill Patients: A Meta-Analysis

**DOI:** 10.3389/fmed.2021.741108

**Published:** 2021-10-12

**Authors:** Jing-Chao Luo, Wen-He Zheng, Chang Meng, Hua Zhou, Yuan Xu, Guo-Wei Tu, Zhe Luo, Hui-Bin Huang

**Affiliations:** ^1^Department of Critical Care Medicine, Zhongshan Hospital, Fudan University, Shanghai, China; ^2^Department of Critical Care Medicine, The Second People's Hospital Affiliated to Fujian University of Traditional Chinese Medicine, Fuzhou, China; ^3^Department of Critical Care Medicine, School of Clinical Medicine, Beijing Tsinghua Changgung Hospital, Tsinghua University, Beijing, China; ^4^Department of Critical Care Medicine, Xiamen Branch, Zhongshan Hospital, Fudan University, Xiamen, China

**Keywords:** cardiopulmonary support, extracorporeal membrane oxygenation, mechanical ventilation, levosimendan, weaning

## Abstract

**Background:** Cardiopulmonary support, as extracorporeal membrane oxygenation (ECMO) or mechanical ventilation (MV), is crucial for ICU patients. However, some of these patients are difficult to wean. Therefore, we aimed to assess the efficacy and safety of levosimendan in facilitating weaning from cardiorespiratory support in this patient population.

**Methods:** We searched for potentially relevant articles in PubMed, Embase, China National Knowledge Infrastructure, Wanfang, and the Cochrane database from inception up to Feb 30, 2021. Studies focusing on weaning data in MV/ECMO adult patients who received levosimendan compared to controls were included. We used the Cochrane risk of bias tool or the Newcastle-Ottawa Quality Assessment Scale to evaluate the study quality. The primary outcome was the weaning rate from MV/ECMO. Secondary outcomes were mortality, duration of MV, and ICU stay. Subgroup analysis, sensitivity analysis, and publication bias were also conducted.

**Results:** Eighteen studies with 2,274 patients were included. The quality of the included studies was low to moderate. Overall, levosimendan effectively improved weaning rates from MV/ECMO [odds ratio (OR) = 2.32; 95%CI, 1.60–3.36; *P* < 0.00001, *I*^2^ = 68%]. Subgroup analyses confirmed the higher successful weaning rates in ventilated patients with low left ventricular ejection fractions (OR = 4.06; 95%CI, 2.16–7.62), patients with ECMO after cardiac surgery (OR = 2.04; 95%CI, 1.25–3.34), and patients with ECMO and cardiogenic shock (OR = 1.98; 95%CI, 1.34–2.91). However, levosimendan showed no beneficial effect on patients with MV weaning difficulty (OR = 2.28; 95%CI, 0.72–7.25). Additionally, no differences were found concerning the secondary outcomes between the groups.

**Conclusions:** Levosimendan therapy significantly increased successful weaning rates in patients with cardiopulmonary support, especially patients with combined cardiac insufficiency. Large-scale, well-designed RCTs will be needed to define the subgroup of patients most likely to benefit from this strategy.

## Introduction

In the intensive care unit (ICU), cardiopulmonary support is the most common and essential therapy. Mechanical ventilation (MV) is a well-established supportive therapy for patients suffering from various forms of respiratory failure (1). Extracorporeal membrane oxygen (ECMO) is increasingly used to treat patients with intractable hypoxemia or circulatory failure (2, 3). However, long-term cardiopulmonary support is not without its risks. Prolonged MV/ECMO can increase the risks of pneumonia, lung injuries, and skeletal muscle atrophy. Delayed weaning is also associated with increased morbidity, mortality, and length of stay in ICU or hospital (4). Therefore, appropriate early weaning from cardiopulmonary support is pretty necessary.

However, some ICU patients are difficult to wean from cardiopulmonary support (5, 6). The weaning failure is related to various causes of diaphragmatic weakness, especially in patients with cardiac or pulmonary comorbidities (7). The weaning procedure increases left ventricular filling pressures and pulmonary artery pressures, and the resulting increased cardiac burden may be one of the main reasons for weaning failure (8).

Levosimendan is a novel positive inotropic drug that effectively treats acute and chronic decompensated heart failure and is becoming used for weaning from cardiopulmonary support in recent years (9–12). Unlike the traditional inotropic drugs, such as epinephrine, dobutamine, or dobutamine, levosimendan increases cardiac output without adding myocardial oxygen consumption (13). Besides, similar to the myocardium, levosimendan can also strengthen the contraction of respiratory muscles, thereby promoting weaning (14).

Several publications have recently emerged on levosimendan use in ICU patients who undergo weaning from MV/ECMO, with discrepancies among the results (10, 12, 15–17). Therefore, we sought to conduct a systematic review and meta-analysis by pooling available studies to investigate the levosimendan's efficacy and safety in ICU patients during MV/ECMO weaning.

## Methods

We performed this systematic review and meta-analysis following the PRISMA guidance (18) (Additional File 1), and our protocol has been registered on the International Platform of Registered Systematic Review and Meta-analysis Protocols database (Registration number: INPLASY 202170024) and is available in full on inplasy.com (https://doi.org/10.37766/inplasy2021.7.0024). Ethical approval was not required for our work.

### Search Strategy

Two authors (J-CL and CM) independently searched for eligible studies in the PubMed, Embase, Cochrane Library database, China National Knowledge Infrastructure, and Wanfang Database before Feb 30, 2021, which was the last search. We limited our language to English and Chinese. Details in the literature search terms were summarized in Additional File 2. The search strategy was restricted to RCTs and observational studies with matched groups (cohort studies with two-arms or case-control studies). We also evaluated the reference lists of relevant studies to ensure the inclusion of all potential studies.

### Study Selection

Studies were assessed for eligibility if they fulfilled the following criteria: (1) comparing levosimendan to control (i.e., placebo, any other drug or no drug) in patients undergoing MV/ECMO; (2) reporting data on the successful weaning rate from MV/ECMO. We excluded studies conducted in pregnant women and studies conducted in review, case reports, or case series.

### Data Extraction and Outcomes

The two authors (CM and J-CL) extracted the data independently on the first author's name, study design (retrospective/prospective, RCT/cohort/case-control), year of publication, inclusion criteria, characteristics (age, male or female, and disease severity), levosimendan and control regimens as well as predefined outcomes. The primary outcome was the ECMO or MV weaning. Secondary outcomes included MV duration, length of stay in ICU, overall mortality at the longest following-up available, and adverse events. Discrepancies were identified and resolved through discussion.

### Quality Assessment

CM and J-CL independently evaluated the methodological quality of the individual studies using the Cochrane risk of bias tool for RCTs (19) and the Newcastle-Ottawa Quality Assessment Scale (20) for case-control/cohort studies. We evaluated publication bias by visually inspecting funnel plots when at least 10 studies were included in this meta-analysis.

### Statistical Analysis

The results from all relevant studies were combined to estimate the pooled odds ratio (OR) and associated 95% confidence intervals (CI) for dichotomous outcomes. As to the continuous outcomes, weighted mean differences (WMD) and 95 % CI were estimated as the effect results if they were measured on the same scale and the difference among the means and standard deviation of these outcomes is not significant, otherwise standardized mean difference (SMD) and 95%CI were used. For studies that reported median with accompanying interquartile range (IQR) as the measure of treatment effect, we estimated the mean from median and standard deviations (SD) from IQR using the methods described in previous studies before data analysis.

We used the *I*^2^ statistic to test the heterogeneity. An *I*^2^ <50% was considered as insignificant heterogeneity, and a fixed-effect model was used, whereas a random-effect model was used in cases of significant heterogeneity (*I*^2^ > 50%) using the Mantel-Haenszel method (21). To test the robustness of the primary outcome and explore the potential influence factors, we conducted sensitivity analyses to investigate the influence of a single study on the overall pooled estimate of each predefined outcome. Additionally, subgroup analysis was performed separately by pooling trials focusing on cardiopulmonary support types (MV or ECMO) and cardiac function (low or preserved ejection fraction) for the predefined outcomes. We performed all analyses using Review Manager, Version 5.3.

## Results

### Searching Results

**Figure 1:**
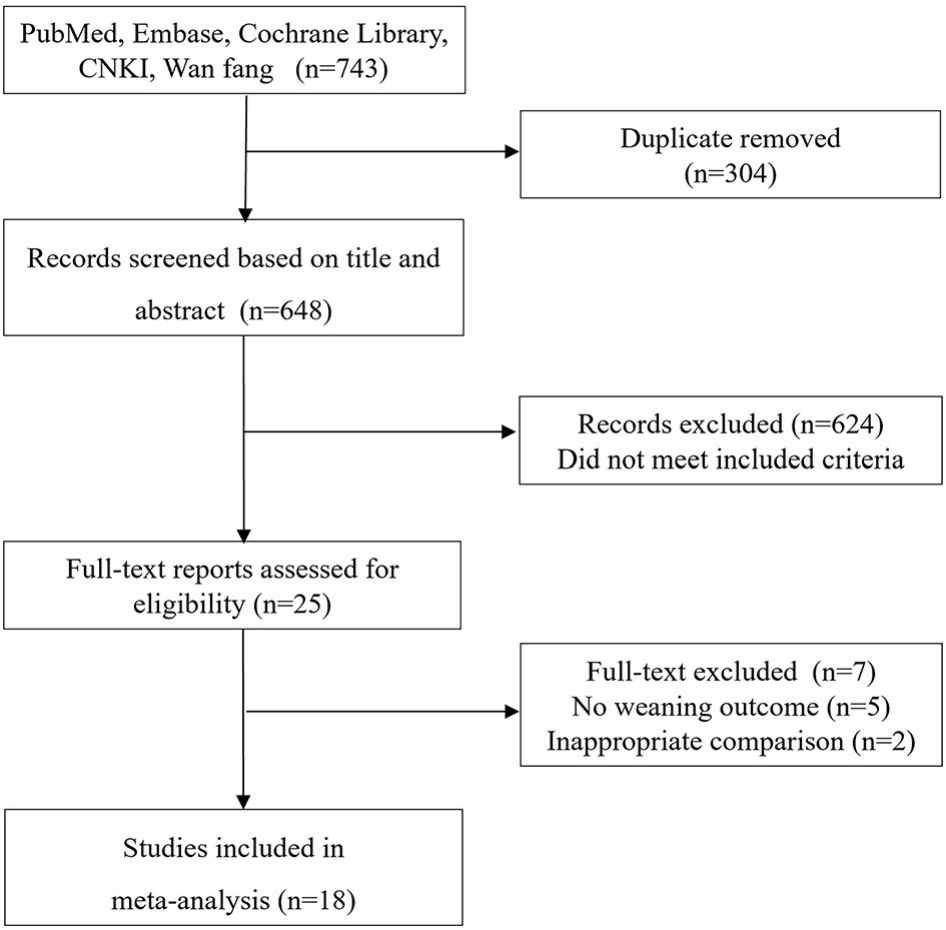
Selection process for studies included in the meta-analysis.

The electronic search yielded 743 records, of which 25 full-text were considered for review. Finally, 18 studies (9–12, 15–17, 22–32) with 2,274 patients met the inclusion criteria and were selected for the final analysis (Figure 1). The details of the search strategy were summarized in Additional File 2.

### Studies Characteristics and Quality Assessment

The main characteristics of included studies and predefined outcome measures are shown in Table 1 and Additional File 3. Fourteen observational studies (9–11, 15–17, 22–26, 30–32) and four RCTs (12, 27–29) were included, which were conducted between 2009 and 2021. All but two studies (12, 28) were single-center studies. Eight (12, 16, 23, 26–29, 31) of the 18 included trials focused on MV weaning, with or without low LVEF of the recruited patients. The remaining ten studies (9–11, 15, 17, 22, 24, 25, 30, 32) focused on ECMO weaning, with five enrolling patients after cardiac surgery (11, 17, 24, 30, 32) and six enrolling patients suffering from cardiogenic shock (9, 10, 15, 22, 25, 32). Most included studies reported the detail of the levosimendan therapy regimen. As to the control group, two studies (24, 27) used milrinone while others used a placebo or no use.

**Table 1:** Characteristics of the included studies.

**Study**	**Study design**	**Population**	**Patient characteristics (Levosimendan group/Control group)**						
			**Sample**	**Mean age (yesrs)**	**Male (%)**	**Disease severity, mean**	**Levosimendan**	**Control**	**Form of support**
Shaker (29)	RCT, SC	Patients of abdominal malignancy, EF <35% and CHF	30/30	62/60	60/73	ASA III: 22/20 ASA IV: 8/10	Infusion at 0.1 μg/kg/min or placebo for 24 h	Infused placebo at 0.1 μg/kg/min for 24 h	MV
Pan (23)	P, SC	Patients of weaning difficulty	50/50	67/67	54/58	NA	Infusion of 12.5 mg for 24 h	None	MV
Huang (27)	RCT, SC	Patients with RF and AHF	30/30	74/69	57/53	NA	Infusion of 12.5 mg for 24 h	Infusion milrinone of 12.5 mg for 24 h × 7 days	MV
Eriksson (28)	RCT, MC	Patients undergoing CABG with impaired LVEF <0.5	30/30	64/64	93/87	Euro-SCORE: 5/5	12 μg/kg bolus, followed by an infusion of 0.2 μg/kg/min	Infused placebo with 12 μg/kg bolus, followed by an infusion of 0.2 μg/kg/min	MV
Vlasova (31)	R, SC	Patients undergoing CABG with low LVEF	17/29	64/60	NA	NA	Infusion of 12.5 mg for 24 h	None	MV
Gordon (12)	RCT, MC	Patients with sepsis	215/218	67/69	NA	APACHE II:25/25 SOFA:10/10	Infusion of 0.05–0.2 μg/kg/min for 24 h	Infusion of placebo at 0.05–0.2 μg/kg/min for 24 h	MV
Chen (16)	P, SC	Patients of weaning difficulty	45/38	NA	NA	NA	Infusion of 12.5 mg for 24 h	None	MV
He (26)	P, SC	Patients of weaning difficulty	37/31	NA	NA	NA	Infusion of 12.5 mg for 24 h	None	MV
Zipfel (25)	R, SC	Patients with refractory cardiogenic shock	37/49	NA	NA	NA	NA	NA	ECMO
Affronti (22)	R, SC	Patients with cardiogenic shock	6/11	57/56	67/63	NA	Infusion of 12.5 mg for 24 h	None	ECMO
Vally (9)	R, SC	Patients with cardiogenic shock	51/99	54/53	71/63	SAPS II: 59.2/55.5	Infusion of 12.5 mg for 24 h	None	ECMO
Distelmaier (30)	R, SC	Patients after cardiac surgery	179/61	65/65	74/63	Euro SCORE 11/9	Infusion of 12.5 mg for 24 h	None	ECMO
Jacky (24)	R, SC	Patients after cardiac surgery	26/38	66/63	81/76	SAPS II: 53/49	Infusion rate of 0.1 mg/kg/h	Infused milrinone at 10 mg/min	ECMO
Kevin (11)	P, SC	Children after cardiac surgery	54/91	0.7/0.96	48/56	NA	12.5 μg/kg bolus; following 0.2 mg/kg/min	None	ECMO
Guilherme (15)	R, SC	Patients with refractory cardiogenic shock	53/147	54/53	62/65	SAPS II: 53.5/51.7 SOFA:11.5/11.8	Infusion of 0.1 μg/kg/min for 1 h; followed 0.1–0.2 μg/kg/min for 24 h	None	ECMO
Deschka (17)	R, SC	Patients after cardiac surgery	78/198	NA	NA	NA	NA	NA	ECMO
Alonso-Fernandez-Gatta (10)	R, SC	Patients with circulatory compromise	23/100	60/62	74/73		Infusion of 12.5 mg with rate of 0.1 μg/kg/min	none	ECMO
Haffner (32)	R, SC	Patients of cardiogenic shock or following cardiotomy	27/36	NA	NA	NA	NA	NA	ECMO

*AHF, acute heart failure; APACHE II, acute physiology and chronic health evaluation II; CABG, Coronary Artery Bypass Grafting; CHF, chronic heart failure; ECMO, Extracorporeal Membrane Oxygenation; Euro SCORE, European System for Cardiac Operative Risk Evaluation; LVEF, left ventricular ejection fraction; MV, mechanical ventilation; MC, multicenter; P, prospective; R, retrospective; SAPS II, simplified acute physiology score II; SC, single center; SOFA, sequential organ failure assessment*.

We evaluated the included studies' risk of bias using the Newcastle-Ottawa Quality Assessment Scale for the 14 observational studies (9–11, 15–17, 22–26, 30–32) and the Cochrane risk-of-bias tool for the four RCTs (12, 27–29) (Additional File 4). The quality of case-control/cohort studies was moderate to high, and the risk of bias in RCTs was low in all critical domains. Assessment of publication bias using visually inspecting funnel plots showed no potential publication bias among the included studies (Additional File 5).

### Primary Outcome

**Figure 2:**
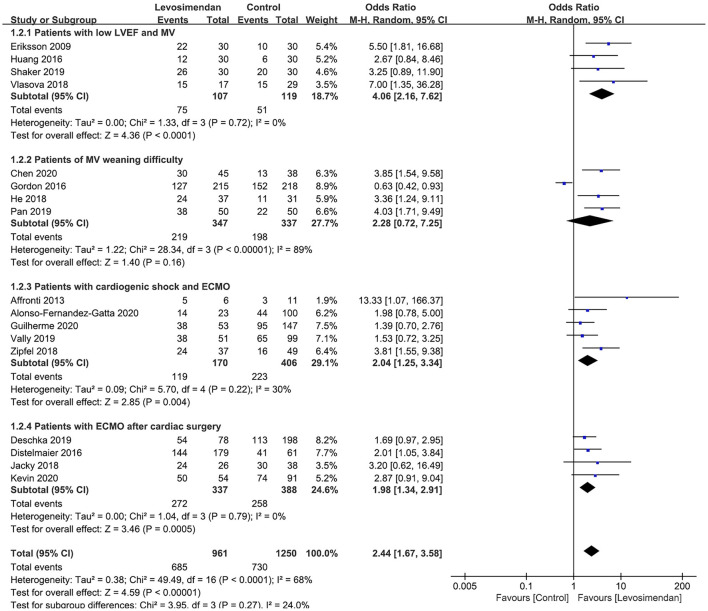
Forest plots of the levosimendan therapy on weaning rates from cardiopulmonary support.

All 18 studies reported MV/ECMO weaning rates. The pooled analysis showed that, compared with control, levosimendan improved MV/ECMO weaning (*n* = 2,274; OR = 2.32; 95%CI, 1.60 to 3.36; *P* < 0.00001), with high heterogeneity (*I*^2^ = 68%) among the studies. In the sensitivity analysis, excluding any single trial did not significantly alter the overall combined OR (*P*-value ranging from <0.00001 to <0.0001). Similarly, subgroup analyses confirmed the higher successful weaning rates in patients with low LVEF and MV (27–29, 31), patients with ECMO after cardiac surgery (11, 17, 24, 30), and patients with ECMO and cardiogenic shock (9, 10, 15, 22, 25) (Figure 2). However, levosimendan showed no beneficial effect on patients with MV weaning difficulty than the control group (12, 16, 23, 26) (Figure 2).

### Secondary Outcomes

**Figure 3:**
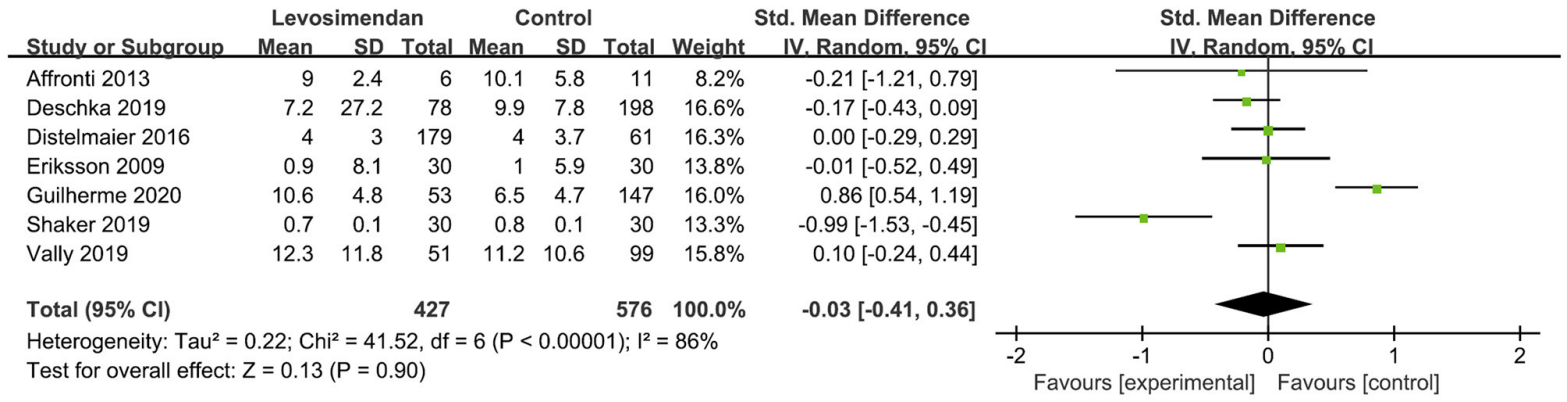
Forest plots of the effects of levosimendan therapy on duration of mechanical ventilation.

**Figure 4:**
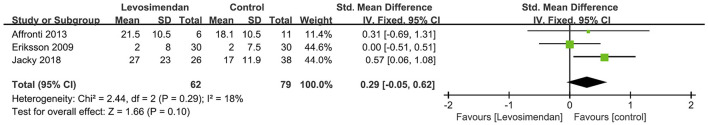
Forest plots of the effects of levosimendan therapy on the length of stay in intensive care unit.

**Figure 5:**
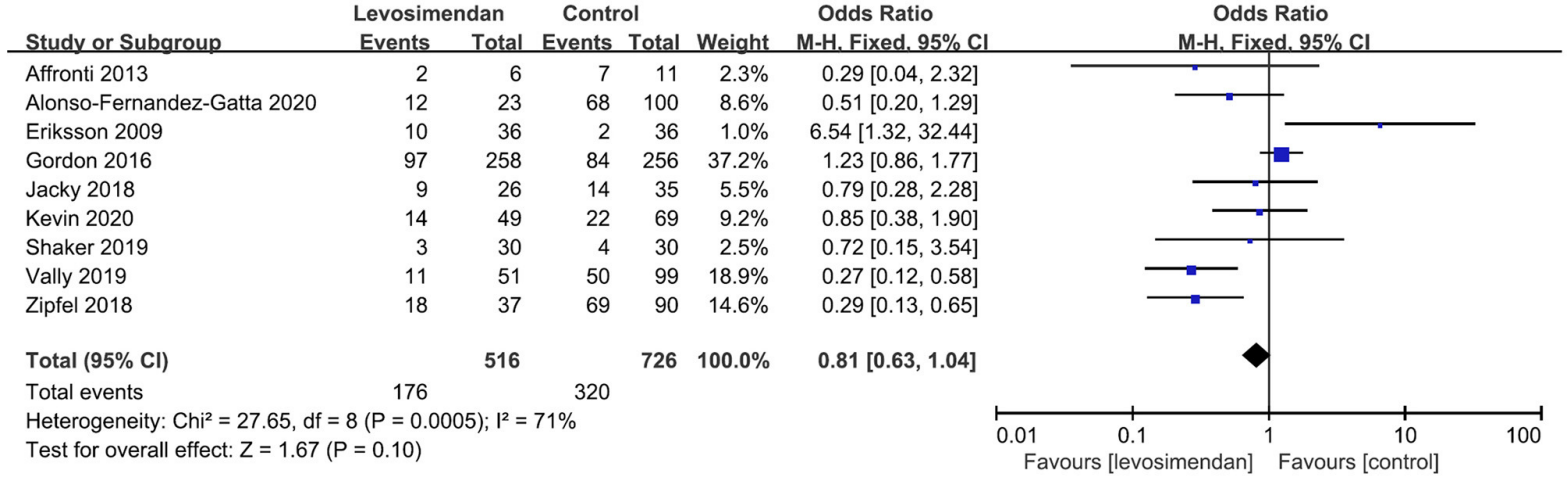
Forest plots of the effects of levosimendan therapy on overall mortality.

There was no significant differences between the levosimendan and control groups in duration of MV/ECMO (s studies, *n* = 1,003; SMD = −0.03 days; 95% CI, −0.41 to 0.36; *I*^2^ = 86%; *P* = 0.90) (9, 15, 17, 22, 28–30) (Figure 3) and length of stay in ICU (3 studies, *n* = 141, SMD = −0.29 days; 95% CI, −0.05 to 0.62, *I*^2^ = 18%; *P* = 0.10) (22, 24, 28) (Figure 4). Nine studies reported specific data on outcome of overall mortality, and pooled results showed no significant difference between the groups (9 studies, *n* = 1,225; OR = 0.81; 95% CI, 0.63–1.04; *I*^2^ = 71%; *P* = 0.10) (9–12, 22, 24, 25, 28, 29) (Figure 5). We further conducted subgroup analyses based on ECMO or MV for the secondary outcomes. We found that the use of levosimendan was associated with a significant reduction in mortality rate in patients receiving ECMO [6 studies, 596 patients, 0.66 (0.53, 0.81), *P* = 0.0001] but not MV therapy. Meanwhile, subgroup analyses also showed no differences in the duration of MV/ECMO, ICU, or hospital LOS between the groups, either in the ECMO patients or in the MV patients. Only three studies (10, 12, 22) reported the advert events summarized in Additional File 6. There was no statistically significant difference in the incidence of complications (ARF requiring RRT, bleeding, ECMO-related complications, pneumonia, bleeding, ischemic stroke, hemorrhagic stroke, tracheostomy, acute liver failure, arrhythmia, myocardial infarction, or acute coronary syndrome) between the groups (all *P* > 0.05).

## Discussion

This study evaluated the effect of levosimendan on successful weaning from MV and ECMO in critically ill patients. The quality of the included studies was low to moderate. The pooled data showed that: (1) Levosimendan effectively improved ICU patients' weaning from VA-ECMO therapy. (2) Levosimendan showed benefits in improving the weaning rate from MV in patients with low LVEF but not in those with preserved LVEF. (3) Subgroup-analyses showed that use of levosimendan was associated with a significant reduction in mortality rate in patients receiving ECMO but not MV therapy. Additionally, no differences were found in other secondary outcomes between groups.

### Levosimendan in Weaning From ECMO

We found levosimendan facilitates the weaning from ECMO and reduces mortality rate, which is consistent with the findings of two previous meta-analyses (33, 34). Both meta-analyses reported levosimendan could improve weaning from ECMO based on five (*N* = 557) (34) and seven (*N* = 630) (33) studies, respectively. Our study added several newly published studies based on the previous meta-analyses with a large sample size of 1,336 patients, which allowed for better statistical efficacy and allowed subgroup analyses to verify our results' robustness.

VA-ECMO is increasingly being used in the short-term management of refractory circulatory failure. The main indications are myocarditis, cardiac arrest, refractory cardiogenic shock, and post-cardiotomy cardiac failure in high-risk patients with reduced LVEF (35, 36). However, this patient population still has high weaning failures. Our results show that ~40% of patients cannot successfully wean from VA-ECMO treatment under the conventional weaning process. Therefore, clinical research concerning improvement in the weaning rate of ECMO is rising (10, 15). After all, a successful weaning is a prerequisite for patient survival.

In the present study, we found that the successful weaning from VA-ECMO based on levosimendan was 80%, significantly higher than that of 60% in the control group. Some properties of levosimendan may explain its benefit for weaning from VA-ECMO. The most important thing is the sensitization of calcium ions, the positive inotropic effect without a significant increase in oxygen consumption in the myocardium (13). Second, levosimendan is an effective vasodilator. By opening ATP-dependent potassium channels in vascular smooth muscle, levosimendan has various protective effects against ischemic myocardium (preconditioning, post-processing, anti-coma, and anti-apoptotic effects) (37). Compared with other cardiotonic drugs, the effect of levosimendan is not affected by the combined use of β-blockers, and it lacks an arrhythmia-promoting effect. In addition, the long-lasting effects of its circulating active metabolites (up to 8–9 days) allow it to allow gradual weaning and provide continuous support during the critical period after ECMO (38).

### Levosimendan in Weaning From MV

About 26–42% of ICU intubated patients have difficulty weaning from MV, increasing morbidity, mortality, and healthcare costs (5, 12, 16, 29). Diaphragm dysfunction is one of the critical factors contributing to weaning failure in such a patient population (7). Currently, no explicit drugs help restore diaphragm function. Therefore, whether levosimendan can improve diaphragm function, as it does in the myocardium, has aroused widespread interest. Some published studies supported such a hypothesis (14, 39). An *in vitro* study (39) showed that by increasing calcium sensitivity, levosimendan could enhance the contractility of diaphragm muscle fibers in patients with or without COPD. In the RCT by Doorduin et al. (14), the authors recruited 30 healthy volunteers who underwent an inspiratory loading task and found that levosimendan significantly improved neuromechanical efficiency and contractile function (*P* < 0.05) than placebo.

However, the results of our meta-analysis of clinical studies did not fully confirm this hypothesis. Patients who gained benefits from levosimendan during MV weaning are still those with concurrent low LVEF (27–29, 31). For such a patient population, cardiac function is the most critical factor of weaning. During the weaning process, abrupt transfer from MV to spontaneous breathing may significantly increase left ventricular filling pressure and pulmonary artery pressure (8). Simultaneously, sympathetic excitation induces the release of catecholamines, leading to peripheral vasoconstriction and increased cardiac workload. These mechanisms can cause heart failure and weaning failure, especially in patients with previous cardiac or pulmonary comorbidities.

In contrast, pooled studies focusing on patients without low LVEF showed no benefits of levosimendan on weaning from MV (12). Some explanations might help understand this failure. Firstly, most studies enrolling patients who already met the criteria for difficult weaning from MV. Besides heart function, the reasons for weaning difficulty in the ICU setting are respiratory, psychological, and psychomotor nutritional, while these factors are often combined to complicate the weaning process (5). Secondly, the patient's disease can influence the effect of levosimendan. As shown in the leoPARD study (12), the authors enrolled patients with sepsis/septic shock without septic myocardial suppression and found levosimendan was associated with a lower weaning rate from MV (HR 0.77, 95%CI 0.60–0.97, *P* = 0.03). The reason may be that levosimendan can cause peripheral vasodilation, resulting in the need for more norepinephrine to maintain blood pressure and cause increased adequate arterial elasticity to increased afterload. Therefore, the mechanicals might diminish the benefit of enhanced myocardial contraction from levosimendan. Moreover, the increased incidence of side effects of levosimendan, such as rapid supraventricular arrhythmias, might also contribute to the weaning failure (12).

Additionally, we found no levosimendan benefit in the length of stay in ICU or hospital. This may be because, for critically ill patients, ICU or hospital discharge was not always determined by the condition of the patients. The hospital policy to accept or refuse critically ill patients in general wards and the availability of beds for patients requiring long-term rehabilitation therapy may affect the length of ICU or hospital stay.

### Study Limitation

Our research has some limitations. First of all, most of the included are retrospective studies, especially studies on ECMO. This greatly affected the causality of our research conclusions. At present, some evaluations aim to assess whether the administration of levosimendan is related to RCT studies that reduce the weaning failure, such as the LEVOECMO trial (NCT04728932), is ongoing. The results of these studies will further verify our conclusions. Second, in the included studies, there is significant heterogeneity in the standard setting of patient weaning and the usage of levosimendan. Third, the study we included did not find any patients treated for VV-ECMO. Finally, the included ICU patients have different underlying diseases. However, due to the number of studies, we cannot conduct a subgroup analysis to clarify this further.

## Conclusion

In summary, based on the current evidence, levosimendan is significantly associated with successful weaning rates from cardiopulmonary support in ICU patients, especially those with a combination of cardiac insufficiency. However, further well-designed RCTs will be needed to define the subgroup of patients most likely to benefit from this strategy.

## Data Availability Statement

The original contributions presented in the study are included in the article/Supplementary Material, further inquiries can be directed to the corresponding author/s.

## Author Contributions

W-HZ and CM searched the scientific literature and drafted the manuscript. J-CL helped to collect the data and performed statistical analyses. HZ, G-WT, and YX participated in the design of the study and performed the statistical analysis. H-BH and ZL contributed to the conception, design, data interpretation, manuscript revision for critical intellectual content, and supervision of the study. All authors read and approved the manuscript.

## Conflict of Interest

The authors declare that the research was conducted in the absence of any commercial or financial relationships that could be construed as a potential conflict of interest.

## Publisher's Note

All claims expressed in this article are solely those of the authors and do not necessarily represent those of their affiliated organizations, or those of the publisher, the editors and the reviewers. Any product that may be evaluated in this article, or claim that may be made by its manufacturer, is not guaranteed or endorsed by the publisher.

## Abbreviations

CI: confidence interval
ECMO: extracorporeal membrane oxygen
ICU: intensive care unit
LVEF: left ventricular ejection fractions
MD: mean difference
MV: mechanical ventilation
RR: risk ratio
RCTs: randomized controlled trials
SD: standard deviations.
