# Supplementary material for: Levosimendan to Facilitate Weaning From Cardiorespiratory Support in Critically Ill Patients: A Meta-Analysis
Source: Front Med (Lausanne). 2021 Oct 12;8:741108. doi: 10.3389/fmed.2021.741108 (PMC8546177; doi:10.3389/fmed.2021.741108)
Supplement: Supplementary file 1 [file Data_Sheet_1.PDF]

Levosimendan to facilitate weaning from cardiorespiratory support in critically ill patients: A meta-analysis

Jing-Chao Luo[2], MD; Wen-He Zheng[2], MD; Chang Meng[1], MD; Hui-Bin Huang[1]*, MD; Hua Zhou[1], MD; Zhe Luo[2]*, MD; Yuan Xu[1], MD

*Corresponding author: 1.Hui-Bin Huang, Email: hhba02922@btch.edu.cn.

2.Zhe Luo, Email: luo.zhe@zs-hospital.sh.cn

Additional files

Additional file 1 PRISMA checklist....2
Additional file 2 Search Strategy....5
Additional file 3 Summary of clinical outcomes reported in the included studies....8
Additional file 4 Assessment of study quality 10
Additional file 5. Funnel plot of comparison: weaning rate....12
Additional file 6: Complications among the included studies....13

Additional file 1 PRISMA 2009 checklist

Section/topic	#	Checklist item	Reported on page #
**TITLE**			
Title	1	Identify the report as a systematic review, meta-analysis, or both.	1
**ABSTRACT**			
Structured summary	2	Provide a structured summary including, as applicable: background; objectives; data sources; study eligibility criteria, participants, and interventions; study appraisal and synthesis methods; results; limitations; conclusions and implications of key findings; systematic review registration number.	3
**INTRODUCTION**			
Rationale	3	Describe the rationale for the review in the context of what is already known.	5
Objectives	4	Provide an explicit statement of questions being addressed with reference to participants, interventions, comparisons, outcomes, and study design (PICOS).	6
**METHODS**			
Protocol and registration	5	Indicate if a review protocol exists, if and where it can be accessed (e.g., Web address), and, if available, provide registration information including registration number.	7
Eligibility criteria	6	Specify study characteristics (e.g., PICOS, length of follow-up) and report characteristics (e.g., years considered, language, publication status) used as criteria for eligibility, giving rationale.	7
Information sources	7	Describe all information sources (e.g., databases with dates of coverage, contact with study authors to identify additional studies) in the search and date last searched.	7
Search	8	Present full electronic search strategy for at least one database, including any limits used, such that it could be repeated.	7 and Appendix 2
Study selection	9	State the process for selecting studies (i.e., screening, eligibility, included in systematic review, and, if applicable, included in the meta-analysis).	7
Data collection process	10	Describe method of data extraction from reports (e.g., piloted forms, independently, in duplicate) and any processes for obtaining and confirming data from investigators.	7-8

Data items	11	List and define all variables for which data were sought (e.g., PICOS, funding sources) and any assumptions and simplifications made.	8
Risk of bias in individual studies	12	Describe methods used for assessing risk of bias of individual studies (including specification of whether this was done at the study or outcome level), and how this information is to be used in any data synthesis.	8
Summary measures	13	State the principal summary measures (e.g., risk ratio, difference in means).	8
Synthesis of results	14	Describe the methods of handling data and combining results of studies, if done, including measures of consistency (e.g., I^2) for each meta-analysis.	8
Risk of bias across studies	15	Specify any assessment of risk of bias that may affect the cumulative evidence (e.g., publication bias, selective reporting within studies).	8
Additional analyses	16	Describe methods of additional analyses (e.g., sensitivity or subgroup analyses, meta-regression), if done, indicating which were pre-specified.	8-9
**RESULTS**			
Study selection	17	Give numbers of studies screened, assessed for eligibility, and included in the review, with reasons for exclusions at each stage, ideally with a flow diagram.	10<br>Appendix 3
Study characteristics	18	For each study, present characteristics for which data were extracted (e.g., study size, PICOS, follow-up period) and provide the citations.	10<br>Table 1
Risk of bias within studies	19	Present data on risk of bias of each study and, if available, any outcome level assessment (see item 12).	10
Results of individual studies	20	For all outcomes considered (benefits or harms), present, for each study: (a) simple summary data for each intervention group (b) effect estimates and confidence intervals, ideally with a forest plot.	10-11
Synthesis of results	21	Present results of each meta-analysis done, including confidence intervals and measures of consistency.	11
Risk of bias across studies	22	Present results of any assessment of risk of bias across studies (see Item 15).	Appendix 4
Additional analysis	23	Give results of additional analyses, if done (e.g., sensitivity or subgroup analyses, meta-regression [see Item 16]).	10-11
**DISCUSSION**			
Summary of evidence	24	Summarize the main findings including the strength of evidence for each main outcome; consider their relevance to key groups (e.g., healthcare providers, users, and policy makers).	12-15

Limitations	25	Discuss limitations at study and outcome level (e.g., risk of bias), and at review-level (e.g., incomplete retrieval of identified research, reporting bias).	15
Conclusions	26	Provide a general interpretation of the results in the context of other evidence, and implications for future research.	16
**FUNDING**			
Funding	27	Describe sources of funding for the systematic review and other support (e.g., supply of data); role of funders for the systematic review.	17

Additional file 2 Search Strategy

Search Strategy

Database: PubMed, Embase, Cochrane library

Search completed 30th Feb 2021

PubMed

((("simendan"[Mesh]) OR (levosimendan[Title/Abstract])) OR (Calcium sensitizer[Title/Abstract])) AND ((((((((((extracorporeal membrane oxygenation[Title/Abstract]) OR (ECMO[Title/Abstract])) OR (extracorporeal life support[Title/Abstract])) OR (ECLS[Title/Abstract])) OR (mechanical circulatory support[Title/Abstract])) OR (mechanical ventilation[Title/Abstract])) OR (ventilated[Title/Abstract])) OR (ventilation[Title/Abstract])) OR (ventilator[Title/Abstract])) AND ((((critical care[Title/Abstract]) OR (critically ill[Title/Abstract])) OR (intensive care[Title/Abstract])) OR ("Critical Care"[Mesh]))))

Embase

#21 #5 AND #10 AND #20

#20 #11 OR #12 OR #13 OR #14 OR #15 OR #16 OR #17 OR #18 OR #19

#19 'ventilator':ab,ti AND [embase]/lim

#18 'ventilated':ab,ti AND [embase]/lim

#17 'ventilation':ab,ti AND [embase]/lim

#16 'mechanical circulatory support':ab,ti AND [embase]/lim

#15 'ecls':ab,ti AND [embase]/lim

#14 'extracorporeal life support':ab,ti AND [embase]/lim

#13 'ecmo':ab,ti AND [embase]/lim

#12 'extracorporeal membrane oxygenation':ab,ti AND [embase]/lim

#11 'extracorporeal oxygenation'/exp

#10 #6 OR #7 OR #8 OR #9

#9 'intensive care'/exp

#8 'intensive care':ab,ti AND [embase]/lim

#7 'critically ill':ab,ti AND [embase]/lim

#6 'critical care':ab,ti AND [embase]/lim

#5 #1 OR #2 OR #3 OR #4

#4 ' calcium sensitizer ':ab,ti AND [embase]/lim

#3 ' simendan '/exp

#2 'levosimendan'/exp

#1 ('levosimendan'/exp OR levosimendan) AND [embase]/lim

Cochrane library

#1 MeSH descriptor: [Extracorporeal Membrane Oxygenation] explode all trees

#2 ("Extracorporeal Membrane Oxygenation "):ti,ab,kw (Word variations have been searched)

#3 (ECMO):ti,ab,kw (Word variations have been searched)

#4 (extracorporeal life support):ti,ab,kw (Word variations have been searched)

#5 (ECLS):ti,ab,kw (Word variations have been searched)

#6 (mechanical circulatory support):ti,ab,kw (Word variations have been searched)

#7 (ventila*):ti,ab,kw (Word variations have been searched)

#8 #1 OR #2 OR #3 OR #4 OR #5 OR #6 OR #7

#9 MeSH descriptor: [Critical Care] explode all trees

#10 MeSH descriptor: [Critical Illness] explode all trees

#11 (critically ill):ti,ab,kw (Word variations have been searched)

#12 (intensive care):ti,ab,kw (Word variations have been searched)

#13 (critical care):ti,ab,kw (Word variations have been searched)

#14 #9 OR #10 OR #11 OR #12 OR #13

#15 (levosimendan):ti,ab,kw (Word variations have been searched)

#16 MeSH descriptor: [Simendan] explode all trees

#17 (calcium sensitizer):ti,ab,kw (Word variations have been searched)

#18 #15 OR #16 OR #17

#19 #8 AND #14 AND #18

Wanfang database

主题:（危重症 or 重症监护 or 重症 or ICU or 呼吸机 or 机械通气 or 体外膜肺 or ECMO）and 主题:（左西孟旦 or 钙离子增敏剂）

[Results: 13]

China National Knowledge Infrastructure database

TKA=(“左西孟旦” + “钙离子增敏剂”) and TKA=("危重症 + “重症监护” + “重症” + “ICU” + “呼吸机” + “机械通气” + “体外膜肺” + “ECMO”)

Studies needed for full-reviewed but not included in the current meta-analysis (n=7 trials)

1. Sterba M, Banerjee A, Mudaliar Y. Prospective observational study of levosimendan and weaning of difficult-to-wean ventilator dependent intensive care patients. Crit Care Resusc. 2008 Sep;10(3):182-6.

2. Sangalli F, Avalli L, Laratta M, Formica F, Maggioni E, Caruso R, Cristina Costa M, Guazzi M, Fumagalli R. Effects of Levosimendan on Endothelial Function and Hemodynamics During Weaning From Veno-Arterial Extracorporeal Life Support. J Cardiothorac Vasc Anesth. 2016 Dec;30(6):1449-1453.

3. Landoni G, Lomivorotov VV, Alvaro G, Lobreglio R, Pisano A, Guarracino F, Calabrò MG, Grigoryev EV, Likhvantsev VV, Salgado-Filho MF, Bianchi A, Pasyuga VV, Baiocchi M, Pappalardo F, Monaco F, Boboshko VA, Abubakirov MN, Amantea B, Lembo R, Brazzi L, Verniero L, Bertini P, Scandroglio AM, Bove T, Belletti A, Michienzi MG, Shukevich DL, Zabelina TS, Bellomo R, Zangrillo A; CHEETAH Study Group. Levosimendan for Hemodynamic Support after Cardiac Surgery. N Engl J Med. 2017 May 25;376(21):2021-2031.

4. Xu CX, Li L, Gong SJ, Yu YH, Yan J. [The effects of levosimendan on the cardiac function and prognosis in elderly patients with septic shock and myocardial contractility impairment]. Zhonghua Nei Ke Za Zhi. 2018 Jun 1;57(6):423-428. Chinese.

5. Wang C, Gong J, Shi S, Wang J, Gao Y, Wang S, Peng YG, Song J, Wang Y. Levosimendan for Pediatric Anomalous Left Coronary Artery From the Pulmonary Artery Undergoing Repair: A Single-Center Experience. Front Pediatr. 2018 Aug 14;6:225.

6. Roesthuis L, van der Hoeven H, Sinderby C, Frenzel T, Ottenheijm C, Brochard L, Doorduin J, Heunks L. Effects of levosimendan on respiratory muscle function in patients weaning from mechanical ventilation. Intensive Care Med. 2019 Oct;45(10):1372-1381.

7. Mauriat P, Bojan M, Soulie S, Foulgoc H, Tafer N, Ouattara A. Impact of the perioperative inotropic support in grown-up congenital heart patients undergoing cardiac surgery: a propensity score adjusted analysis. Ann Intensive Care. 2020 Jul 9;10(1):91.

Appendix 3. Summary of clinical outcomes reported in the included studies

Study	LVEF% (Lev/Ctrl)	Duration of MV/ECMO (Lev/Ctrl)	LOS (Lev/Ctrl)		Weaning success (Lev/Ctrl)	Mortality(%) (Lev/Ctrl)
			ICU	Hospital		
Shaker 2019[29]	28/27	16.5±2.3h/ 19.2±2.5h	NA	8.5±2.3d/ 12.6±4.5d	(26/30)/(20/30)	90d:(3/30)/(4/30)
Pan 2019[23]	NA	NA	NA	NA	(38/50)/(22/50)	NA
Huang 2016[27]	37/38	NA	NA	NA	(12/30)/(6/30)	NA
Eriksson 2009[28]	36/36	21 (10-792) h/ 23 (10-580) h	2 (1-33) d/ 2(1-31) d	NA	(22/30)/(10/30)	In hospital: (10/36)/(2/36)
Vlasova 2018[31]	35/35	NA	NA	NA	(15/17)/(15/29)	NA
Gordon 2016[12]	NA	NA	NA	NA	(127/215)/(152/218)	In hospital: (97/258)/ (84/256)
Chen 2020[16]	NA	NA	NA	NA	(30/45)/(13/38)	NA
He 2018[26]	NA	NA	NA	NA	(24/37)/(11/31)	NA
Zipfel 2018[25]	NA	182.4 h/216 h	NA	NA	(24/37)/(16/49)	In hospital: (18/37)/(69/90)
Affronti 2013[22]	16/17	9±2.4d/ 10.1±5.8	21.5±10.5d/ 18.1±8.5d	32.8±18.7d/ 29.7±18d	(5/6)/(3/11)	In hospital: (2/6)/(7/11)
Vally 2019[9]	19/20	12.3±11.8d/ 11.2±10.6d	NA	NA	(38/51)/(65/99)	30d:(11/51)/(50/99)
Distelmaier 2016[30]	NA	4±2.96d/ 4±3.70d	NA	NA	(144/179)/(41/61)	NA
Jacky 2018[24]	NA	NA	27±23.0d/ 17±11.9d	33±24.4d/ 22±14.8d	(24/26)/(30/38)	28d:35/40 180d:50/44
Kevin 2020[11]	15/30	NA	NA	NA	(50/54)/(74/91)	In hospital:29/32
Guilherme 2020[15]	NA	10.6 ± 4.8d/ 6.5 ± 4.7 d	NA	NA	(38/53)/(95/147)	NA
Deschka 2019[17]	NA	7.2± 27.2d/ 9.9±7.8d	NA	NA	(54/78)/(113/198)	NA
Alonso-Fernandez-	18/31	NA	NA	NA	(14/23)/(44/100)	In hospital: (12/23)/(68/100)

Gatta 2021[10]						
Haffner 2018[32]	NA	NA	NA	NA	(21/27)/(29/36)	NA

Ctrl=control group, LOS=length of stay, Lev=levosimendan group, NA=not available, Results are expressed as mean ± SD or number.

Additional file 4

Figure S1. Risk of bias graph: review authors' judgements about each risk of bias item presented as percentages across all included studies.

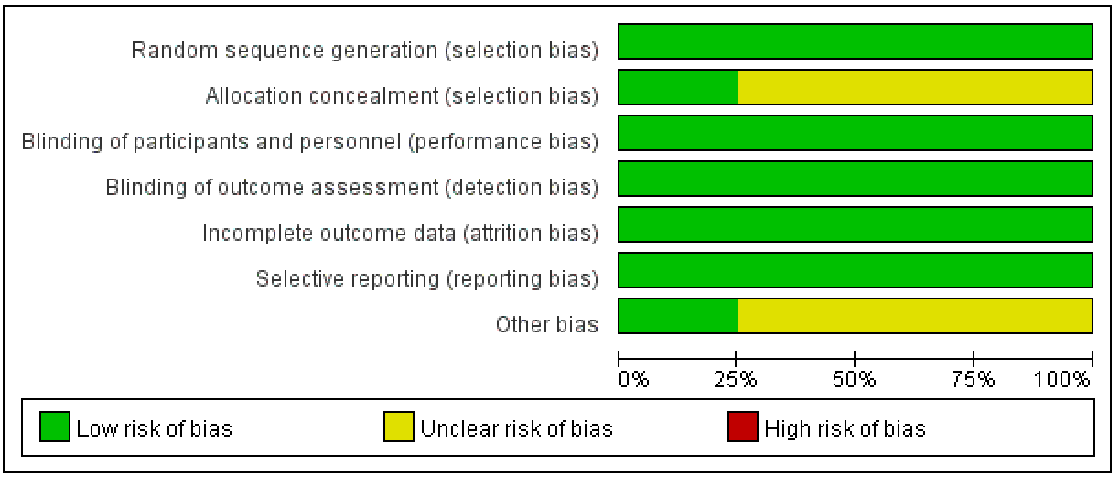

Figure S2. Risk of bias summary: review authors' judgements about each risk of bias item for each included study.

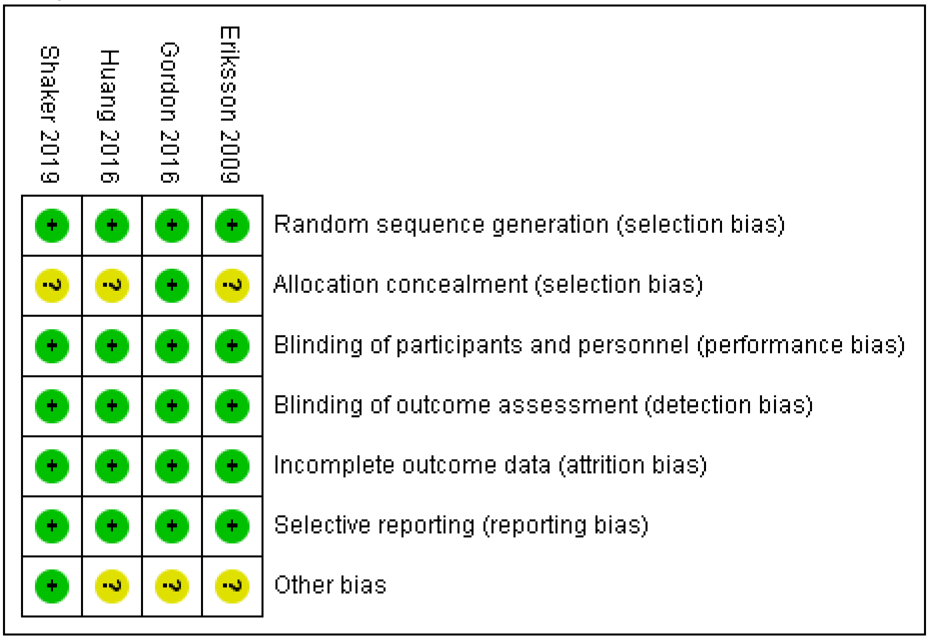

Additional file 4

Risk of bias assessment of the observational studies

Study	Selection				Comparability	Outcome			Total Score
	Exposed Cohort	Non-exposed Cohort	Ascertainment of Exposure	Outcome of Interest		Assessment of Outcome	Length of Follow-up	Adequacy of Follow-up	
Pan 2019[23]	★	★	★	★	☆	★	★	★	7
Vlasova 2018[31]	★	★	★	☆	☆	★	★	★	6
Chen 2020[16]	★	★	★	★	☆	★	★	★	7
He 2018[26]	★	★	★	★	☆	★	★	★	7
Zipfel 2018[25]	★	★	★	☆	☆	★	★	★	6
Affronti 2013[22]	★	★	★	☆	☆	★	★	★	6
Vally 2019[9]	★	★	★	☆	★	★	★	★	7
Distelmaier 2016[30]	★	★	★	☆	★	★	★	★	7
Jacky 2018[24]	★	☆	★	☆	☆	★	★	★	5
Kevin 2020[11]	★	★	★	★	★	★	★	★	8
Guilherme 2020[15]	★	★	★	☆	★	★	★	★	7
Deschka 2019[17]	★	★	★	☆	☆	★	★	★	6
Alonso-Fernandez-Gatta 2021[10]	★	★	★	☆	★	★	★	★	7
Haffner 2018[32]	★	★	★	☆	☆	★	★	★	6

Additional file 5. Funnel plot of comparison: weaning rate

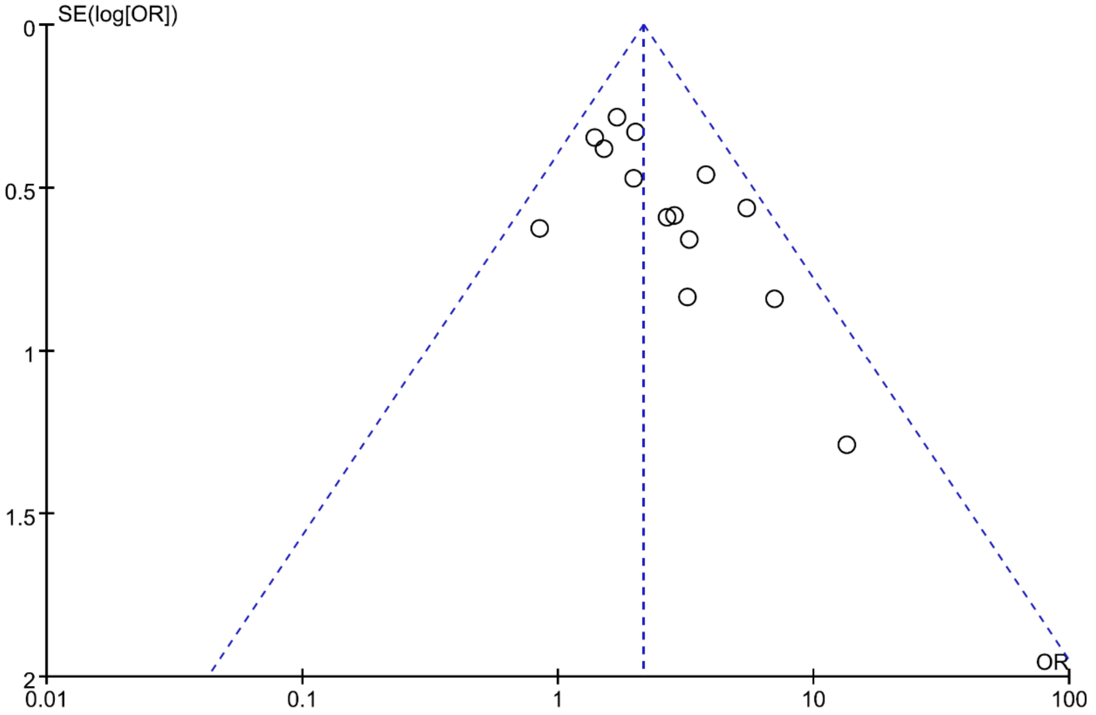

Additional file 6: Complications among the included studies

Complications	Study/year			OR, 95% CI	*p* Value	I^2
ARF requiring RRT	Affronti 2013[22], Alonso-Fernandez 2020[10]	0/6 5/23	4/11 29/100	0.65 (0.24-1.77)	0.4	0%
ECMO-related complications						
Oxygenator failure	Affronti 2013[22]	1/6	3/11		NS	
Surgical site bleeding	Affronti 2013[22]	2/6	4/11		NS	
Surgical site infection	Affronti 2013[22]	1/6	4/11		NS	
Ab ingestis pneumonia	Affronti 2013[22]	1/6	-		NS	
Bleeding	Alonso-Fernandez 2020[10]	15/23	43/100		0.136	
Infections	Alonso-Fernandez 2020[10]	13/23	36/100		0.338	
Ischemic stroke	Alonso-Fernandez 2020[10]	1/23	5/100		0.852	
Hemorrhagic stroke	Alonso-Fernandez 2020[10]	1/23	3/100		0.761	
Tracheostomy	Alonso-Fernandez 2020[10]	5/23	19/100		0.519	
Acute liver failure	Alonso-Fernandez 2020[10]	2/23	10/100		0.795	
Any life-threatening arrhythmia	Gordon 2016[12]	15/258	6/257		0.08	
Myocardial infarction or acute coronary syndrome	Gordon 2016[12]	3/258	1/257		0.62	
Any serious adverse event	Gordon 2016[12]	32/258	23/257		0.26	
